# Random field image representations speed up binary discrimination of brain scans and estimate a phenotype glioblastoma cancer cell model

**DOI:** 10.21203/rs.3.rs-6641557/v1

**Published:** 2025-05-22

**Authors:** William D. ONeill, Julian Najera, Meenal Datta

## Abstract

MRI brain scans alone are not a definitive measure of dementia. Deep-learning algorithms (DLA) and professional human opinion are necessary for diagnosis. Yet, sample sizes are prohibitively large to train a typical DLA, which itself takes considerable computation time to produce diagnostically useful information from contrasting image features. We introduce analytic simplifications to this process to speed it up and reduce data requirements by modeling individual images as solutions of spatially autoregressive (AR) partial difference equations. Image features are the unique individual image AR parameters. Spatially lagged image pixels are explanatory variables for estimating a random-field representation (RFR) of the proposed AR difference equation. RFR model parameters are also those of the image autocorrelation function (ACF). An image pixel matrix-to-vector transformation allows AR parameters to be estimated by ordinary least squares (OLS) regression in millisecond time. Regression degrees of freedom (DOF) -- the number of image pixels -- are unusually large, leading to remarkably precise estimates of AR model parameters. These estimates support a solution of the binary dementia-normal classification of MRI axial brain scans (ADNI and OASIS archives). They also support the AR-RFR process applied to an original microscopic image of a glioblastoma cancer cell. In the face of formidable noise, a sharply defined and robust cancer cell model is estimated, which is an essential tool for cancer - type discrimination exercises and is parametrically plastic enough to serve a wide range of cells.

Autoregressive image models

Image autocorrelation

Rapid dementia discrimination

Cancer cell phenotype

### 1. INTRODUCTION

### 1.1 Binary Neuroimage Classification

Positing a linear, stationary partial difference equation model of a neurological image of human brain might appear demeaning of the organ that defines our superiority over other species, but if mathematics can promote neurological understanding, the inglorious assumption is worth it [1]. Such a model can be an antidote for ambiguous model interpretations and obscure statistical outcomes which accompany some deep learning experiments. Interpretation of deep learning results has been a particularly persistent and nagging complaint of deep learning users [2],[3]. Typical deep learning breast cancer classification applications require convolutional neural networks (CNN) to segment regions of interest for implementing binary classification rules [4]. The current paper avoids deep learning networks by introducing a random field image representation of an image as a finite difference equation whose structure immediately lends itself to statistical support of binary class discrimination using a Fisher Linear Discriminant [5].

The AR parameters of the random field representation are those of the image model autocorrelation function and are used to form a subject's linear discriminant, a scalar product with the Fisher Linear Discriminant eigenvector.

Deep learning methods applied to brain scan images for binary classification of dementia have been modestly successful in special circumstances when large, dedicated computer resources and training data capable of realizing deep learning techniques are available [6]. Yet, discriminator training that requires substantial computer resources and computing time are frequent complaints of deep learning users [7]. The use of deep convolutional neural networks (DCNN), for example, requires models with many parameters and training data sets rarely available to clinical users [8]. The method we describe below is an alternative to some deep learning methods of the binary and higher order classification problems. The binary method is readily expandable to higher order classifications; we shall reproduce later a figure of a ternary example from [9]. Important applications in early diagnosis are especially relevant [10]. In keeping with the need for expanding applications for clinics not having expansive computer resources or large databases, our methodology is applied to small populations requiring only a modest PC or laptop computer. In addition, a random field representation of an image also provides decisive statistical measures for evaluating model parameter estimates and overall model validity [11].

### 1.2 Cancer Cell Image Modeling

Cancer cell image modeling for discrimination requires a very efficient means for treating image noise since cell model differences are frequently slight [12]. In addition, discrimination of cell model comparisons by types can introduce new noise per type [13].

Published attempts seeking a cancer cell discriminator use either training and testing individual cell experiments, [14],[15], or cell discrimination images,[16],[17]. Evered is unique in that cell-pair learning by 150 subjects is the training effort. For the image-based trials literature the training features extracted are typically chemical biomarkers or irregular carcinoma cell patterns.  Computer resources and time are considerable as Support Vector Machine  or Convolutional Neural Networks are the feature extraction algorithms used. Examination of circulating tumor cell dynamics indicates that such  models should obviously be explainable for clinical applications but also there must be an understanding of cancer cell model plasticity that could support cellular-based cancer treatments [18].

It is encouraging that we have found a simple three parameter image model, sharply defined by the order of the difference equation the model represents, for glioblastoma cancer cells.

### 2. METHODS

#### 2.1 Partial Difference Equation Image Models

The utility of partial difference equation image models rests on the observation that a gray scale image in two spatial dimensions is mathematically indistinguishable from a sample function of a gray scale partial difference equation in two dimensions. This image-equation morphism is not new [19],[20]. What is new is our application of an image autocorrelation model of spatially discrete image pixels to estimate the parameters of an AR image model, rather than the estimation of regional image autocorrelations to directly define and refine image properties [21]. In addition, the model we take to define the image autocorrelation is a two-dimensional expanded, spatially discrete, difference equation explaining advection-diffusion dynamics in biological tissue [22].

Three-dimensional numerical solutions have also appeared in the literature [23].

The autocorrelation function of an image random-field representation relates how every image pixel is correlated with every other pixel in the representation. This is a unique defining feature of an image in a random-field representation. Autoregressive parameter estimates are uniquely interpretable as constituents of the discrete, spatial representation of the image autocorrelation function and conversely. Using image autocorrelation functions directly for disease diagnosis does not require explicit individual image modeling [24]. A majority of references to autocorrelation usage in image investigations report methods to detect and minimize autocorrelation found in image attribute interpretations [25].

### 2.2 A Working Example

We consider the following example of how an assumed partial difference equation generates an image random-field representation whose parameters are readily estimable by OLS regression. The difference equation with spatial lags k and l also determines a discrete spatial image model autocorrelation function ρ(k,l).

This working example is readily extended to the general linear and stationary partial difference equation case.

Let $u(x_i,y_j) = u_{i,j}$ be the image pixel intensity at discrete coordinates $(x_i\ y_j)$. We assume the image model is in temporal equilibrium so, $u_{i,j,t} \equiv u_{i,j,t-1}$ for all t. We postulate the image pixel intensity satisfies a linear autoregressive partial difference equation,

$$u_{i,j} = \beta_{10}u_{i-1,j} + \beta_{01}u_{i,j-1} + \beta_{11}u_{i-1,j-1} + \varepsilon_{i,j} \qquad (1)$$

where $\varepsilon_{i,j}$ is zero expected value white noise. In (1), *diffusion* dynamic properties are represented by the mixed index differences and *advection* by the single index differences. If $u_{i,j}$ data is particularly noisy in a given application, $\varepsilon_{i,j}$ can be replaced by an estimable noise model to increase parameter estimation significance.

If the deterministic form of (1), $\varepsilon_{i,j} \equiv 0$, is stable then the discrete AR form in (1) yields a discrete space, autoregressive random-field representation model of $u_{i,j}$

[26]. The autocorrelation function of $u_{i,j}$ satisfies the general definition

$$\rho(k,l) = E[(u_{i,j} - \mu)(u_{i-k,j-l} - \mu)]/\sigma^2 \qquad (2)$$

where $E(u_{i,j}) = \mu$, variance$(u_{i,j}) = \sigma^2$ are expected values with respect to the assumed stationary two-dimensional probability density function of $u_{i,j}$.

A later section of this paper presents a numerical example of (1) that satisfies $\rho(k,l) = \beta_{10}^{|k|}\,\beta_{01}^{|l|}$ for k+l > 0.

### 2.3 Matrix to Vector Transformation

The model in (1) suggests a method of estimating image model parameters $\beta_{i,j}$ from image data. From (1) it is clear $u_{i,j}$ is a weighted sum of $u$ at spatial lags i-1, j ; i,j-1 and i-

1,j-1. Clearly, (1) is the special case p=q=1 of the general autoregressive formula valid for $k+l>0$,

$$u_{i,j} = \sum_{k=0}^{p} \sum_{l=0}^{q} \beta_{k,l}\, u_{i-k,j-l} + \varepsilon_{i,j}. \qquad (3)$$

This equation has q(p+1) + p parameters to estimate.

Estimating partial difference equation parameters is not a simple task even when the equation is stationary and linear [27]. The following development, however, solves this estimation problem for a random-field representation in the linear model case. We illustrate it for (1) wherein it will be obvious that (3) follows the same procedure for general p and q.

Let $v(u_{i,j})$ be a vector of the stacked pixel columns of $u_{i,j}$ over the ranges of i and j, with the first column of $u_{i,j}$ at the top of $v(u_{i,j})$. Since (1) must have elements compatible for matrix addition, i and j ranges must adjust for the spatial lags. For example, if $u_{i,j}$ is m by n then, $2 \leq i \leq m$, $2 \leq j \leq n$ is the range for $u_{i,j}$ , $1 \leq i \leq m - 1$, $2 \leq j \leq n$ for $u_{i-1,j}$ and $1 \leq i \leq m - 1$, $1 \leq j \leq n - 1$ for $u_{i-1,j-1}$.

For scalars $d_1$, $d_2$ and matrices $A_1$, $A_2$ the following is well known [28],

$$v(d_1A_1 + d_2A_2) = d_1v(A_1) + d_2v(A_2). \qquad (4)$$

Apply this to (1) and get

$$v(u_{i,j}) = \beta_{10}\, v(u_{i-1,j}) + \beta_{01}\, v(u_{i,j-1}) + \beta_{11}\, v(u_{i-1,j-1}) + v(\varepsilon_{i,j}), \qquad (5)$$

which is seen to be a vector-matrix population equation

$$x_0 = \beta_{10}\, x_1 + \beta_{01} x_2 + \beta_{11} x_3 + e. \qquad (6)$$

If $b_{ij}$ is the least squares estimate of $\beta_{ij}$ and X is defined as $[x_1\ x_2\ x_3]$ then (6) implies the estimates

$$b = (X^T X)^{-1} X^T x_0, \qquad (7)$$

minimize $e^T e$ for $\det(X^T X) \neq 0$.

It is evident from (7) that b exists if and only if the $x_i$ are linearly independent.

### 2.4 Linear Discriminants of Image Models

The problem of separating (discriminating) normal neurological images from demented ones using a Fisher Linear Discriminant is an eigenvalue problem cast in covariance matrices [29].

Let $B_n$ and $B_d$ be m by k of matrices of respective $b_{ij}$ least square estimates. The $b_{ij}$ elements are estimates of m images, each with k least squares estimated parameters. If $B_n$, $B_d$, have covariance matrices $G_n$ , $G_d$ and $G_p$ is defined as $cov([B_n\ B_d]^T)$, that is, $G_p$ is the covariance matrix of the stacked B matrices, then the Fisher Linear Discriminant eigenvector identity for normally distributed $B_n$ and $B_d$ satisfies [30],

$$[G_p-(G_n+G_d)]v = \lambda\ (G_n+G_d)v \qquad (8)$$

and solution of (8) for eigenvector v of the nonzero eigenvalue $\lambda$ maximally separates the discriminant sample distributions of $z_n=B_nv$ and $z_d=B_dv$. As noted earlier, $B_n$ and $B_d$ entries are estimated with large DOF least squares regressions so those entries can be expected to be normally distributed. The $B_n$ and $B_d$ matrix entries all pass a Kolmogorov-Smirnov normality test at the 0.05 level for the ADNI and OASIS images. **Figure 1** is an additional CDF check for normality of a typical ADNI column of $B_d$ [11].

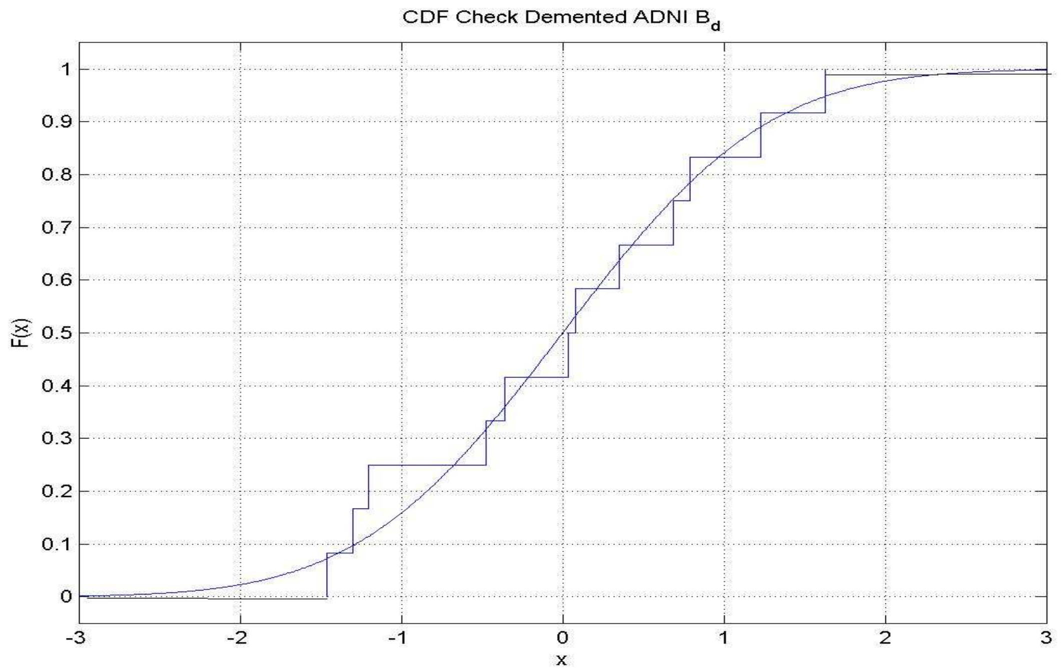

**Figure 1. CDF normality check of a typical ADNI demented parameter vector estimate.**

The $B_n$ and $B_d$ matrices have nornally distributed entries so their projections by the discriminant eigenvector scalar product must be normally distributed; thus, discriminants $z_n=B_nv$ and $z_d=B_dv$ are normally distributed. In addition, $B_n$ and $B_d$ image model parameter matrices are seen to meet the distribution requirements of (8).The test of the discrimination potential of the image AR model parameters is in the separation of the $z_n$ and $z_d$ sample probability distributions.

Thus far, the image modeling considered has been confined to gray scale images, but the theory applies equally as well to RGB or truecolor images by considering each of the truecolor components separately and combining the individual model results for a final truecolor model.

## 3. Results

### 3.1 Parameter Estimation Method Validation

The OLS format of the vector-matrix population equation (6) is the key to rapid and statistically significant parameter estimation for the image models we propose, so it is appropriate to validate the result of these regressions with a known population regression model. The model in (1) is iterated over i and j in [2,200] for $\beta_{01} = 0.92$, $\beta_{10} = 0.96$, $\beta_{11} = - 0.8832$, $\varepsilon_{i.j}$ is zero mean, standard deviation 0.2, Gaussian white noise and all initial conditions are zero. The β parameters chosen,

$\beta_{11} = -\beta_{01}\beta_{10},$ assure that (1) is stable [31]. The statistics of the regression are in **Table 1** where w is a non-parametric statistic defined by,

$$\Pr[b(1\text{-}w/2) \leq \beta \leq b(1 + w/2)] = 0.95 \qquad (9)$$

and b is an OLS estimate of the true β [29].

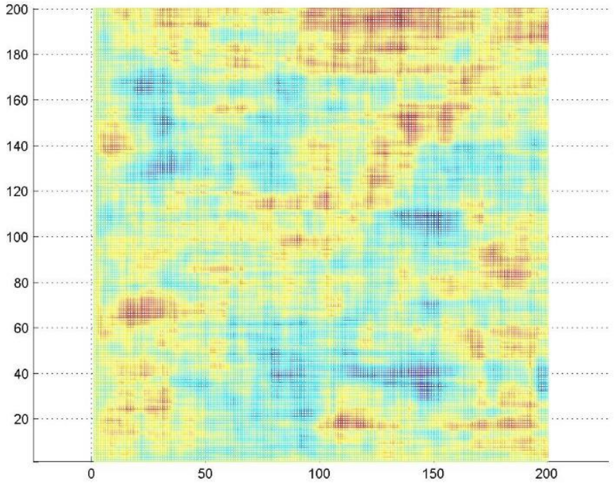

Figure 2

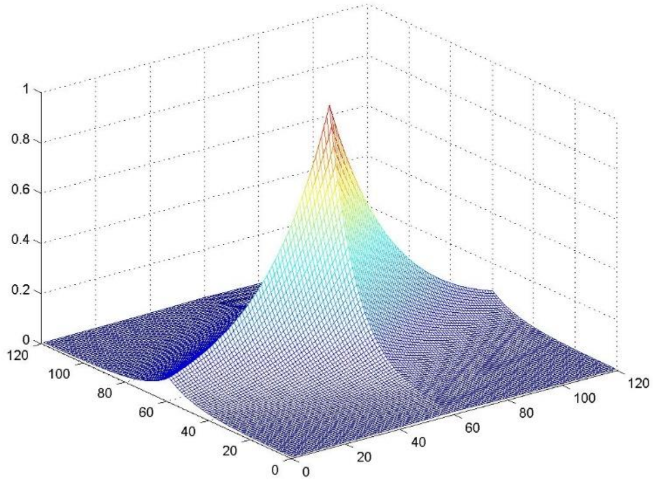

Figure 2A

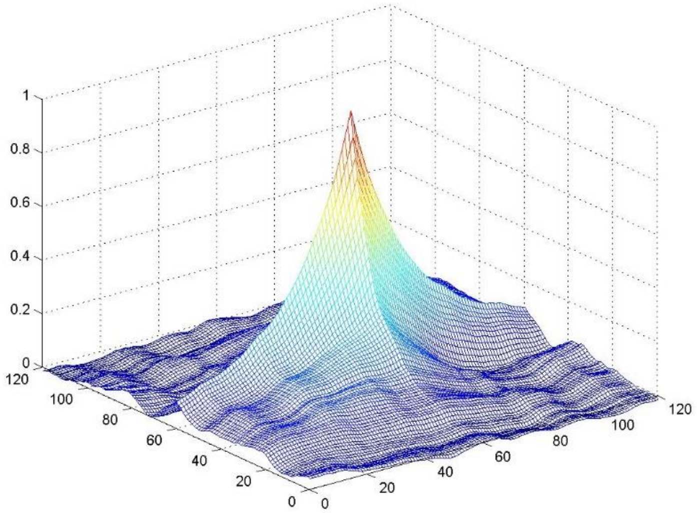

Figure 2B

**Figure 2. 2:Sample function solution of (1). 2A: Autocorrelation function of image 2 using definition (2). 2B: Estimated autocorrelation function of image 2 using Matlab realization of definition (2).**

| | | | |
|---|---|---|---|
| β<br>k,l | 0.96<br>1,0 | 0.92<br>0,1 | -0.8832<br>1,1 |
| b | 0.9623 | 0.9219 | -0.8878 |
| STD(b) | 0.0071 | 0.0099 | 0.0119 |
| t(b) | 135.5 | 93.3 | -74.6 |
| w(b)/2 | 0.033 | 0.048 | 0.059 |

**Table 1. Three parameter OLS model of Figure 2. Degrees of freedom (DOF) = 39601 pixels. $R^2$=0.9862, t(b) = Student-*t* ratios, w(b) is defined by (9) and std(e) = 0.193**

These results, although a simulation, are typical of many OLS applications for image identification, that is, large DOF (pixels) per estimated model parameter [32],[33]. **Table 1** exhibits precisely determined parameters as measured by Student-*t* ratios and w(b)/2 statistics. These results suggest that even modest ‘region of interest’ neurological images, say 50 by 50 pixels, have a generous 500 samples per estimated parameter in 5 parameter models.

The residuals of the validating regression in **Table 1** are Gaussian white noise by design so the t(b) row in **Table 1** is validated. However, it is rare that Student-*t* ratios are valid for real image model OLS estimates since image regression residuals are typically autocorrelated and do not always test from the same distribution (heteroskedastic residuals). These statistical constraints are addressed in the following section.

### 3.2 ADNI Image Models

When p=q=2 in (3) there are 8 model parameters to estimate $b_{ij}$ , i=0,1,2; j=0,1,2, i+j > 0. For an ADNI randomly chosen *normal* subject and an ADNI randomly chosen *demented* subject; axial brain scans are shown in **Figure 3.** The OLS model in (6) is expanded to 8 explanatory columns for this exercise. For the ADNI images there were no significant parameters beyond 8. **Table 2** has the statistics of the image regressions that produced the models in **Figure 3**. The White asymptotic least squares parameter covariance matrix estimate is used to compute standard deviations in **Table 2** as there are 42672 degrees of freedom in the regression and 5334 samples per estimated parameter which meet the asymptotic constraints of the White statistic [34]. This validates Student-*t* statistics in **Table 2.**

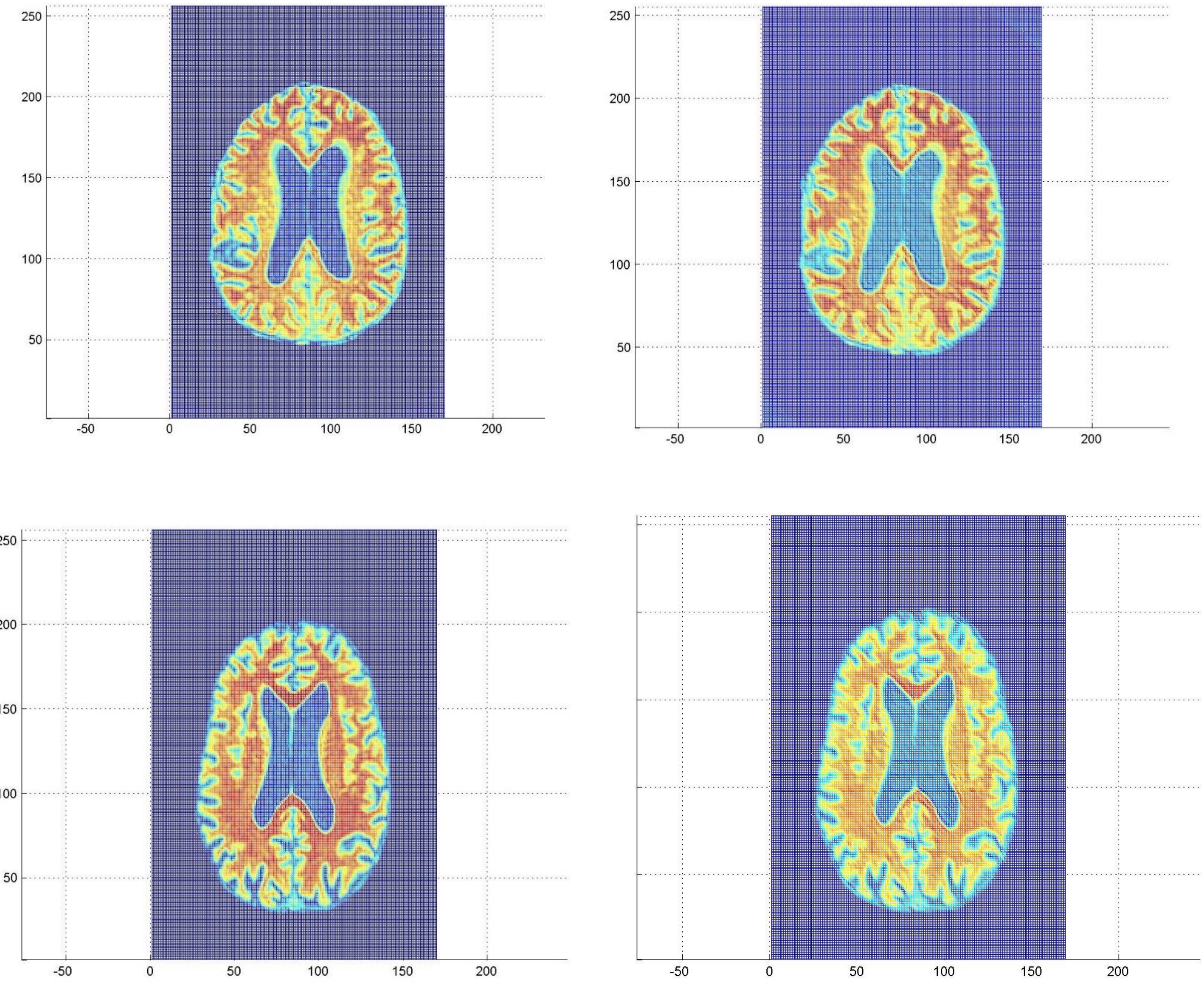

**Figure 3.** ***Top*****: Demented Subject,** ***Bottom*****: Normal subject.** ***Left*****: Original images.** ***Right*****: Ordinary Least Square models of ADNI images. Eight parameter autoregressive models.**

*Demented Subject*

| | $b_{01}$ | $b_{10}$ | $b_{11}$ | $b_{02}$ | $b_{20}$ | $b_{12}$ | $b_{21}$ | $b_{22}$ |
|---|---|---|---|---|---|---|---|---|
| Estimates | 0.9023 | 1.1614 | -0.8628 | -0.2583 | -0.3624 | 0.2271 | 0.2343 | -0.0486 |
| Std($b_{ij}$) | 0.0136 | 0.0122 | 0.0233 | 0.0121 | 0.0123 | 0.0114 | 0.0168 | 0.0117 |
| t- Ratio | 66.2 | 95.5 | -37.1 | -21.3 | -31.8 | 19.9 | 13.9 | -4.1 |
| w(b)/2 | 0.068 | 0.046 | 0.1205 | 0.2098 | 0.1406 | 0.2247 | 0.3217 | 1.091 |
| n=42672 pixels | $R^2$=0.9903 | | | | | | | |

*Normal Subject*

| | $b_{01}$ | $b_{10}$ | $b_{11}$ | $b_{02}$ | $b_{20}$ | $b_{12}$ | $b_{21}$ | $b_{22}$ |
|---|---|---|---|---|---|---|---|---|
| Estimates | 0.8491 | 1.2525 | -0.8797 | -0.1938 | -0.4319 | 0.1560 | 0.2672 | -0.0248 |
| Std($b_{ij}$) | 0.0126 | 0.0109 | 0.0212 | 0.0113 | 0.0102 | 0.0178 | 0.0140 | 0.0101 |
| t- Ratio | 67.2 | 114.6 | -41.5 | -17.2 | -42.4 | 8.78 | 19.1 | -2.45 |
| w(b)/2 | 0.0665 | 0.039 | 0.1075 | 0.26 | 0.1055 | 0.5093 | 0.234 | 1.825 |
| n=42672 pixels | $R^2$=0.9939 | | | | | | | |

**Table 2. Autoregressive model parameter estimates and statistics for ADNI image models in Figure 3.**

The regression results in **Table 2** were computed in 153 milliseconds with design matrices having 126,285 entries. **Figure 4** is an illustrative scatter diagram for a demented subject regression in **Table 2**.

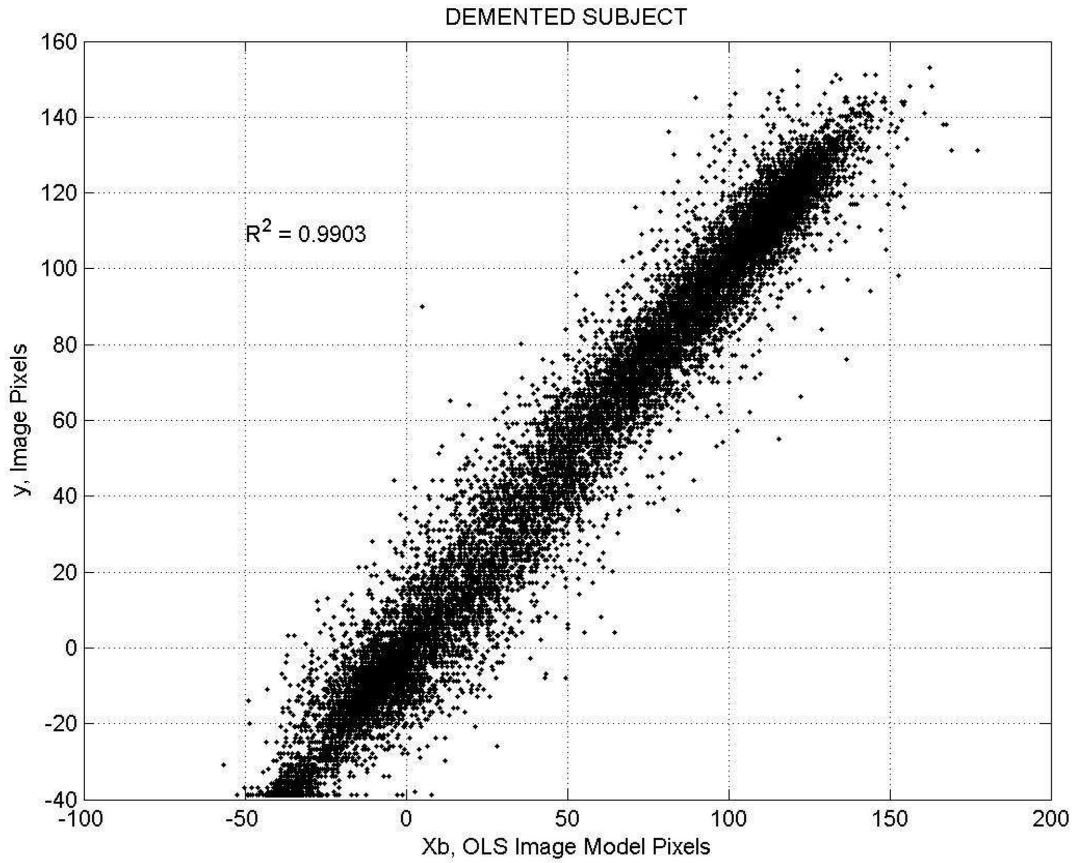

**Figure 4. ADNI Image Regression Scatter Diagram**

### 3.3 OASIS Image Models

Randomly selected OASIS models and statistics are in **Figure 5** and **Table 3**. There were no significant parameters beyond 8 for the OASIS images.

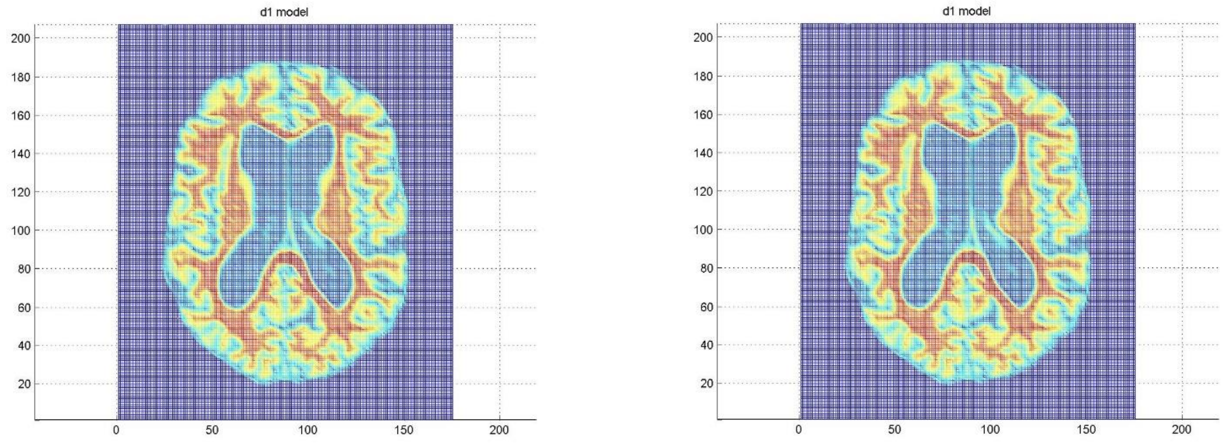

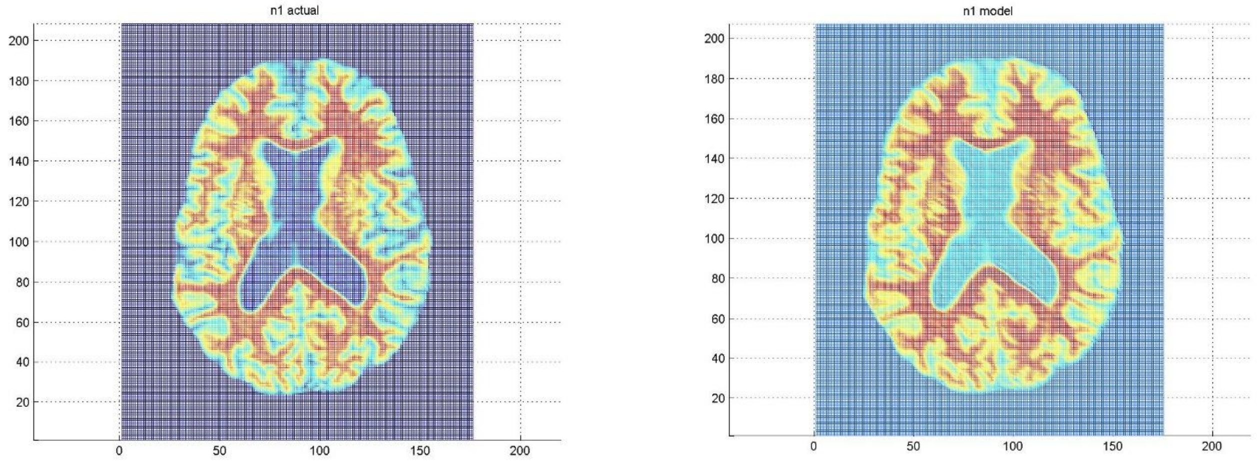

**Figure 5. *Top*: Demented Subject, *Bottom*: Normal Subject. *Left*: Original images. *Right*: Eight parameter autoregressive OASIS model images.**

*Demented Subject*

| | $b_{01}$ | $b_{10}$ | $b_{11}$ | $b_{02}$ | $b_{20}$ | $b_{12}$ | $b_{21}$ | $b_{22}$ |
|---|---|---|---|---|---|---|---|---|
| Estimates | 1.1267 | 0.9899 | -0.9060 | -0.3799 | -0.30 | 0.2727 | 0.2262 | -0.0398 |
| Std($b_{ij}$) | 0.0107 | 0.0109 | 0.0201 | 0.0098 | 0.0101 | 0.0154 | 0.0162 | 0.010 |
| t- Ratio | 105.6 | 95.5 | -47.9 | -38.9 | -29.81 | 17.7 | 14.0 | -3.97 |
| w(b)/2 | 0.042 | 0.047 | 0.093 | 0.1149 | 0.150 | 0.253 | 0.319 | 1.126 |
| n=35844 pixels | $R^2$=0.9929 | | | | | | | |

*Normal Subject*

| | $b_{01}$ | $b_{10}$ | $b_{11}$ | $b_{02}$ | $b_{20}$ | $b_{12}$ | $b_{21}$ | $b_{22}$ |
|---|---|---|---|---|---|---|---|---|
| Estimates | 1.1336 | 1.0445 | -0.9652 | -0.1938 | -0.4319 | 0.1560 | 0.2672 | -0.0248 |
| Std($b_{ij}$) | 0.0126 | 0.0109 | 0.0212 | 0.0113 | 0.0102 | 0.0178 | 0.0140 | 0.0101 |
| t- Ratio | 67.2 | 114.6 | -41.6 | -17.2 | -42.4 | 8.78 | 19.1 | -2.45 |
| w(b)/2 | 0.0665 | 0.0390 | 0.1075 | 0.260 | 0.1055 | 0.5093 | 0.2341 | 1.8253 |
| n=35844 pixels | $R^2$=0.9941 | | | | | | | |

**Table3. Autoregressive eight parameter model estimates and statistics for OASIS image models in Figure 5.**

### 3.4 Linear Discriminants

The Fisher linear discriminator equation (8) can be solved for the eigenvector v given the estimated parameter covariance matrices $G_n$ and $G_d$ of the ADNI images. The ADNI z projection distributions are the lower figure of **Figure 6**. The OASIS $z_n = B_n v$,   $z_d = B_d v$ projection distributions are the top figure of **Figure 6.** The figures indicate 87.5 percent correct classification for the OASIS images and 95.8 for ADNI images. All OASIS and ADNI estimated model parameters passed the Kolmogorov-Smirnov normality test at the 0.05 percent level. Also, as seen in **Figure 1**, typical normal CDF checks confirm the normality of the ADNI and OASIS estimated parameters.

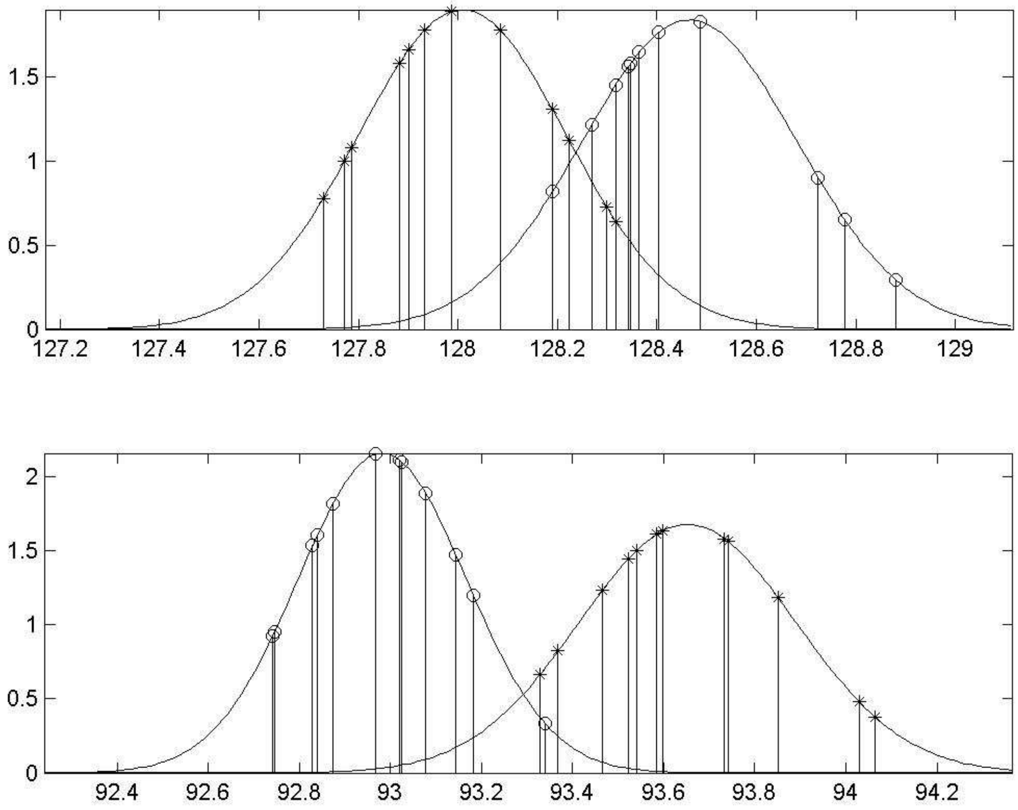

**Figure 6. Discriminant projections for 12 subjects per class, 8 parameter models. Top: OASIS images, Bottom: 12 randomly selected ADNI images per class. Circles are for normal images.**

The distributions in **Figure 6** indicate AR image models have significant image discrimination information in their model parameters.

### 3.5 Equality of Means Hypothesis Test

The $B_n$ and $B_d$ parameter matrices hold the decisive numbers characterizing the modeled images and we have shown these matrices are normally distributed. $G_n$ and $G_d$ are the respective covariance matrices of $B_n$ and $B_d$. Let $m_n$ and $m_d$ be sample mean estimates of $B_n$ and $B_d$. Since $B_n$ and $B_d$ parameter matrices are normally distributed it is possible to test the hypothesis that the means $m_n$ and $m_d$ are equal and therefore the mean discrimination is null. The test simultaneously measures asymptotically how variable the B matrices are and their contribution to the quadratic form of the test through their respective covariances.

The mean *discrimination information* , I(1:2), satisfies the quadratic form [35],

$$I(1{:}2) = (m_n - m_d)^T(G_n/12 + G_d/12)^{-1}(m_n - m_d). \qquad (10)$$

In addition, I(1:2) is asymptotically chi-square distributed with degrees of freedom 6 for 3 parameter models, 16 for 8 parameter models, and 30 for 15 parameter models. The null hypothesis for (10) is $m_n = m_d$; equality of means implies I(1:2) = 0,

**Table 4** summarizes the result of appying the equality of means test to $B_n$ and $B_d$ from ADNI images for spatial lags of 1, 2 and 3. Equality of means is considered not rejected if the cumulative distribution function F(I) of I(1:2) exceeds $\varepsilon = 1 - F(I) = .05$.

| Number of Parameters | 3 | 8 | 15 |
|---|---|---|---|
| Degrees of Freedom | 6 | 16 | 30 |
| I(1:2) | 1.5653 | 28.097 | 47.8417 |
| ε | 0.955 | 0.031 | 0.021 |

**Table 4. The equality of means test for ADNI images of 1, 2, and 3 spatial lags. Equality of means test is not rejected for 3 parameter models.**

### 3.6 Equality of Covariance Matrices Hypothesis

$B_n$ and $B_d$ are normally distributed with covariance matrices $G_n$ and $G_d$ and define $G = (G_n + G_d)/2$ then the *divergence* is

$$J(1:2) = 12\{\log[\det(G)/\det(G_n)] + \log[\det(G)/\det(G_d)]\} \quad (11)$$

and is asymptotically distributed chi-square with k(k+1)/2 degrees of freedom, where k is the number of parameters in the models producing the $G_n$ and $G_d$ covariance matrices; [35].

| Number of Parameters . | 3 | 8 | 15 |
|---|---|---|---|
| J(1:2) | 8.015 | 55.49 | 295.7 |
| ε | 0.237 | 0.021 | 0.00023 |

**Table 5. Equality of ADNI image model covariance matrices hypothesis is not rejected for 3 parameter models**.

The equality of means and covariance matrices tests for 3 parameters (one spatial lag) indicate one spatial lag is not adequate, while the equality tests for 2 and 3 spatial lags emphatically reject the equality null hypotheses. The results in **Tables 4** and **5** also indicate I(1:2) and J(1:2) increase monotonically with the number of model parameters.

I(1:2) and J(1:2) applied to the OASIS image models are given in **Table 6** and **Table 7.** Results are comparable to those of ADNI images, **Table 4**, **Table 5.**

| Number of Parameters | 3 | 8 | 15 |
|---|---|---|---|
| Degrees of Freedom | 6 | 16 | 30 |
| I(1:2) | 0.6898 | 27.55 | 51.29 |
| ε | 0.995 | 0.0358 | 0.0091 |

**Table 6. The equality of means test for OASIS images**

| Number of Parameters | 3 | 8 | 15 |
| --- | --- | --- | --- |
| Degrees of Freedom | 6 | 36 | 120 |
| J(1:2) | 6.4348 | 66.81 | 301.66 |
| ε | 0.3769 | 0.00136 | 0.00026 |

**Table 7. OASIS covariance matrix equality hypothesis.**

### 3.6 Single Cell Image Model

We subject a glioblastoma cancer cell, a primary brain cancer, to an AR-RFR modeling effort since this cancer has been found to have parametric similarities preserved over cell types [36]. The modeling effort demonstrates that while axial and coronal brain scans can frequently produce low noise images compared to other neurological images, other images can be sharply different in their signal to noise ratios and required number of parameters. In particular the glioblastoma image in **Figure 7** and the statistics of **Table 8** suggest we have seen axial and coronal brain scan models can require more than three parameter models, but the glioblastoma image model rejects more than three parameters. The model of **Figure 7** has one spatial lag (3 parameters), and its estimated parameters are in **Table 8** where they are compared to a 5- parameter model of the same cell. Unlike the AR-RFR models of ADNI and OASIS axial brain scans, the three parameter model in **Figure 7** is superior to the five-parameter model in every measure. It is clear from **Figure 8** that the glioblastoma image is significantly more noisy than the ADNI or OASIS scans owing mainly to the magnification required to make the cell visible. However, the degrees of freedom offered by the numerous image pixels are enough to overcome the noise

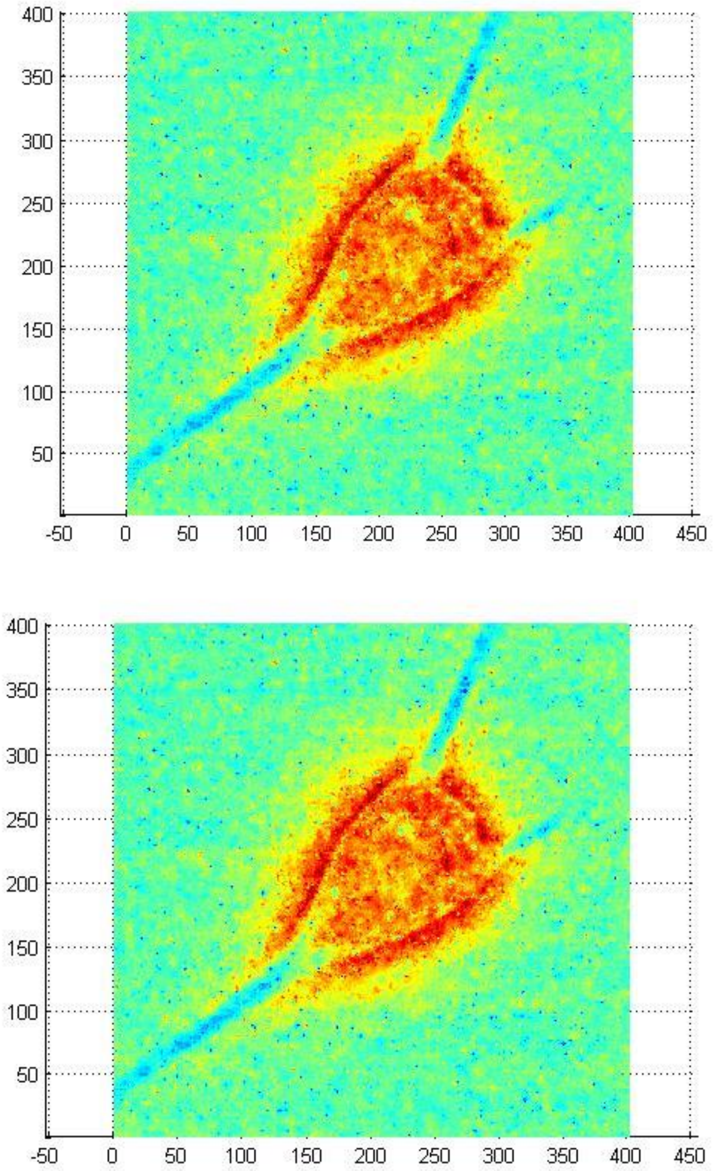

**Figure 7. Top: Original glioblastoma cell image. Bottom: Three parameter OLS glioblastoma cell image model.**

and allow significant parameters to be estimated.

| | $b_{01}$ | $b_{10}$ | $b_{11}$ | $b_{02}$ | $b_{20}$ |
|---|---|---|---|---|---|
| Estimates | 0.5606 | 0.6012 | -.1373 | -.0473 | -.0034 |
| Std($b_{ij}$) | .0053 | .0055 | .0044 | .0039 | .0036 |
| t-Ratio | 105.8 | 109.3 | -31.2 | -16.9 | -0.94 |
| w(b)/2 | .042 | .037 | 0.143 | 0.368 | 4.22 |
| n=160000 pixels | $R^2$ =0.91 | | | | |
| Estimates | 0.5574 | 0.5679 | -.149 | | |
| Std($b_{ij}$) | .0041 | .004 | .0045 | | |
| t-Ratio | 135.9 | 141.9 | -33.1 | | |
| w(b)/2 | .033 | .031 | 0.13 | | |
| n=160000 pixels | $R^2$ =0.92 | | | | |

**Table 8. Three parameter and five parameter glioblastoma models**

The OLS regressions have DOFs of 160,000 pixels which preserve the parameter estimate precision while making the 3 parameter model more efficient than the 5 parameter model.

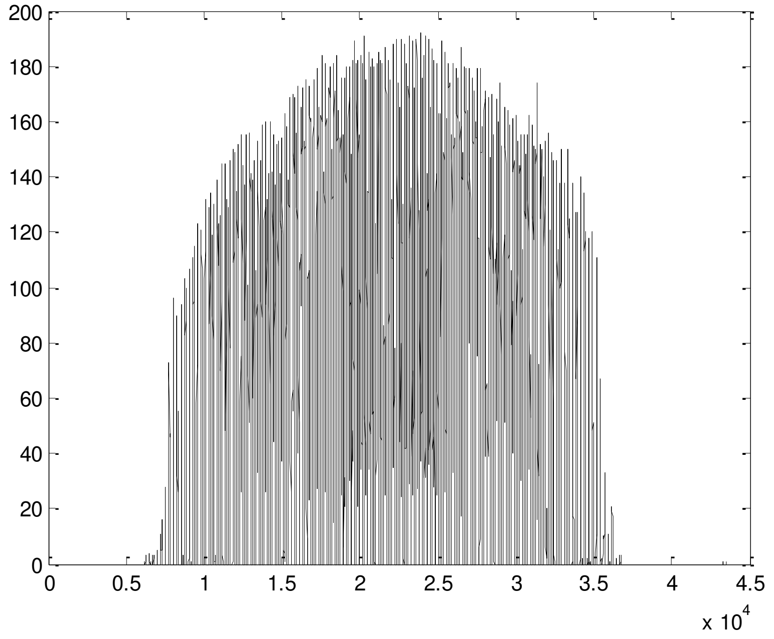

200
180
160
140
120
100
80
60
40
20
0
0
0.5
1
1.5
2
2.5
3
3.5
4
4.5
x 10^4

522

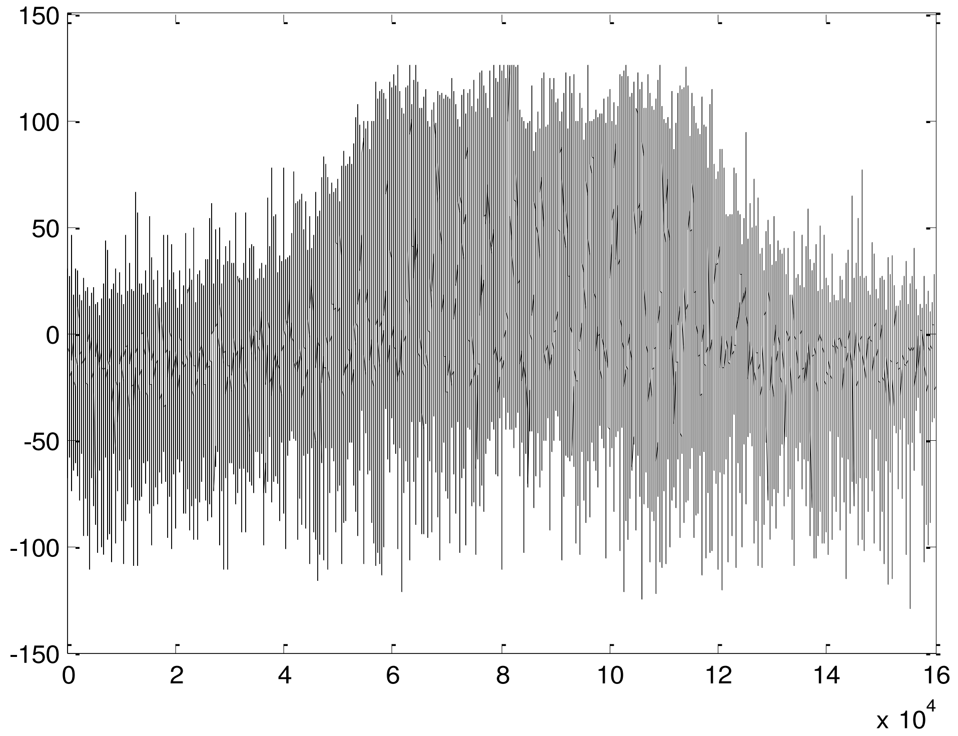

150
100
50
0
-50
-100
-150
0
2
4
6
8
10
12
14
16
x 10^4

523

**Figure 8. Top: Vector form of an axial MRI pixel column. Bottom: Vector form of a central glioblastoma pixel column.**

## 4. Future Studies

The binary class discrimination exercise is frequently seen as an approximation to a higher order discrimination problem, for example, radiologists frequently consider binary dementia classification an approximation to a ternary normal-demented- mild cognitive impairment, MCI, classification. Ternary classification then can track the progress of MCI potentially becoming one of the seven recognized dementias [29].

The following ternary discrimination example from [9] is illustrative of the general case and proposes a possible research investigation in higher order discriminations.

Let classes one and two be murine sarcoma and murine carcinoma mammary tumors respectively. Class three will be human carcinoma mammary tumors. We intuitively expect class 3 will have linear discriminants separated farther from classes 1 and 2 than the latter from each other. Parameter covariance matrices are $G_1$ and $G_2$ for murine subjects, $G_3$ for human and $G_4$ for the stacked parameter matrices. The two projecting nonzero eigenvectors in v , their nonzero eigenvalues $\lambda$, satisfy the eigenvector- eigenvalue equation,

$$(G_4-G_1-G_2-G_3)v = \lambda(G_1+G_2+G_3)v. \qquad (12)$$

The eigenvector projections are indicated in **Figure 9** from [9], which confirm the intuitive relative locations. The projections in **Figure 9** were computed in 2.07 seconds.

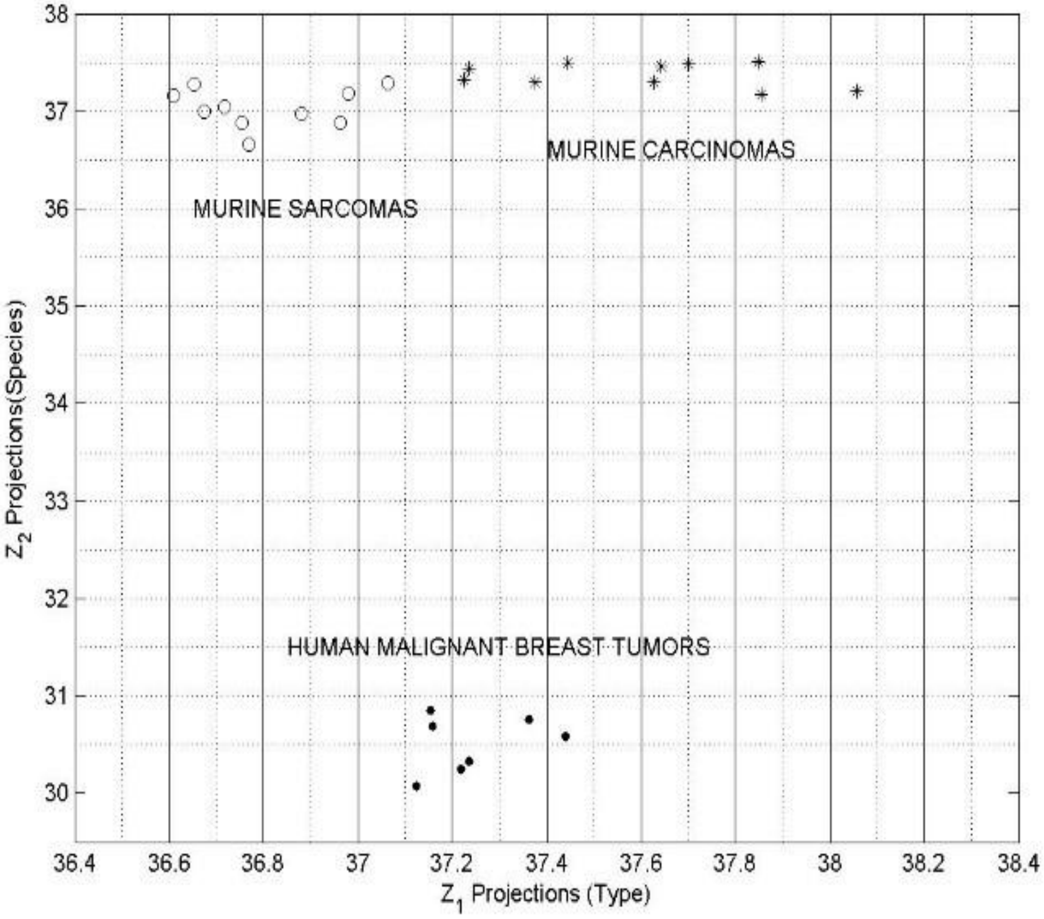

**Table 9. Three class discrimination projections: Two murine mammary tumor types and one human female mammary tumor type.**

## 5. Neurological Image Sources

### 5.1 <u>Axial MRI Images</u>

We use a randomly selected subset of axial MRI images from a previous study having 97 subjects [29]. The ADNI archive axial MR image slices were 1.5 T, T1 weighted using the MP Rage sequence, calibration scans for $B_1$ correction, dual fast spin echo and were 256 by 170 gray scale pixels. All ADNI images were selected from one slice axially above the anterior commissure brain structure. ADNI subject inclusion criteria varied over four classes: (I) normal subjects had no subjective memory complaints; (II) normal memory function stratified by educational attainment; (III) MMSE scores between 24 and 30; and (IV) a Clinical Dementia Rating (CDR) of 0. Early Mild Cognitive Impairment (EMCI) subjects had subjective memory complaints, abnormal memory function stratified by age, MMSE 24 to 30, a CDR of 0.5, and AD could not be diagnosed at time of screening visit. Late Mild Cognitive Impairment (LMCI) subjects had the same criteria as the EMCI subjects, but educational attainment was lower. AD subjects had memory complaints, abnormal memory function, MMSE scores 20 to 27, CDR of 0.5 to 1.0, and satisfied a National Institute of Neurological Disorders and Stroke (NINCDS) criteria for probable AD. The subjects we took as normal were screened as males, ages 65 to 84 years with MMSE scores of 29 to 30. The subjects we took as demented were of ages 65 to

84 years with MMSE scores of 17 to 27. Therefore, subjects were identified as normal or demented according to the range of their MMSE scores. It is likely that about 70% of the categorized demented subjects suffered from AD [29]. All ADNI axial images had cranium and dura artifacts manually cropped.

The OASIS axial MR image slices were screened as right-handed males of ages 65 to 75 years with MMSE scores of 29 to 30. The database produced 14 such images called the normal class. Screening for right-handed males of ages 67 to 84 years with MMSE scores of 17 to 27 produced 12 images we refer to as the demented class. These images came from a set of 416 subjects aged 18 to 96. For each subject, 3 or 4 individual T1-weighted MRI scans obtained in single scan sessions were averaged to get the images, 208 by 176 pixels, for our normal subjects. OASIS images had dura and cranium artifacts manually cropped. All axial images from the OASIS archive were at a fixed, unspecified number of 1.5 mm slices from the anterior commissure brain structure.

4.2 Single Cell Culture

Commercially available immortalized human cell lines were used in the cell experiments. U87 glioblastoma (GBM) cells (American Type Culture Collection [ATCC], Manassas, VA, USA) were cultured at 37 degrees C in a humidified 5% $CO_2$ atmosphere, as in our previous study[36]. Cells were maintained in Dulbecco's Modified Eagle Medium (Invitrogen, Carlsbad, CA, USA) supplemented with 10% fetal bovine serum (Gibco,

Grand Island, NY, USA) and1X penicillin-streptomycin (Invitrogen, Carlsbad, CA, USA).

Cells were seeded in surface treated T-tissue culture flasks (VWR, Radnor, PA, USA) at a density of ~1.6-2.2 x 103 cells/cm2 and allowed to grow for 48 hours. This low seeding density was chosen to ensure that images of single cells could be taken over multiple days.

### 4.3 <u>Single Cell Imaging</u>

Phase contrast images of single cells were collected over a period of 3 days with a Leica DMi1 Inverted Phase Contrast Microscope equipped with the

FLEXACAM C1 camera (Leica Microsystems, Deerfield, IL, USA). Images were specifically taken at 20x magnification using a HI PLAN I objective. At this magnification, the micron:pixel ratio is 0.15. For each image, the brightness was adjusted to ensure that light was not obfuscating the cell body and an outline of the cell could be easily discerned [36].

## 5. Conclusions

### 5.1 Contributions

The assumption that a two-dimensional neuroimage is a solution of a two-dimensional linear, stationary partial difference equation is a readily testable hypothesis. The discrete-space form of the partial difference equation, after undergoing a linear vectorizing transformation, presents a rigorous statistical model-- a Random Field Representation --for testing assorted hypotheses of image models. Its solution has the desirable property that image model parameters are unambiguously those of the image model's autocorrelation function.

In the modeling process, validation of image random field hypotheses  resulted in a necessary and sufficient condition for the existence of a linear autoregressive image model. This result suggests only degenerate images would not satisfy the condition. The ordinary least squares image parameter estimation problem, solvable on a millisecond time base, is a hypothesis with robust statistical support in the form of *degrees of freedom* equal to the number of image pixels. As noted, even for spatially diminutive ‘region of interest’ investigations, large degrees of freedom are available for significant parameter estimation when image pixels are explanatory variables. Eight parameter image models--two spatial lags-- adequately reject mean and covariance equality and the models are typically estimated in 5 milliseconds or less. This timing implies 60 images per second videos could readily track class image parameter estimates in real time. Tracking neurological image parameter estimates in real time would be interesting when the images are functional cognitive retinal images [37].

The estimated image parameters are sufficient in solving the binary image classification problem with a linear discriminant.  A Fisher Linear Discriminant applied to a random-field representation of image models produces satisfactory classifications of images from two image archives for models having more than one spatial lag. Computation times are less than three seconds for the 24 models estimated.

The Fisher Linear Discriminant results also have been shown to be true for subject images with ternary classes  and we include a discriminant projection plot from that study to show how the ternary projections can be interpreted as signals of class change and/or particular class therapeutic progress [9].

The partial difference equation of a random-field representation supports image parameters well- defined as constituting the random-field representation autocorrelation function. This property alone can be of crucial importance in some applications [38].

### 5.2 Limitations

It is evident from the examples presented that finding an optimal model (mean square sense) for a particular image is a guessing game in the p and q integer space constrained by a need to model minimum error variance while accepting only significantly estimated parameters to do so. The  equality of means hypothesis and equality of covariance matrices hypothesis are useful for solving these problems but more typical is the problem of excessive image noise. ‘Excessive’ here means the modeling effort fails because noise levels prevent significant parameters from being estimated. In such a case an image noise model can be simultaneously estimated, for example, by adding

$$c_{01}\,\varepsilon_{i,j-1} + c_{10}\,\varepsilon_{i-1,j} \qquad (13)$$

to (1) to make it an autoregressive-moving average --ARMA -- image model. If $c_{01}$ and $c_{10}$ are significantly estimated, then the $b_{i,j}$ are likely to be also.

Mathematical descriptions of neurological images by deep learning methods have an enviable history of accomplishments but also well described limitations, not the least of which is the need for larger computer resources if deep learning methods are to continue to innovate in structural MRI studies and simultaneously serve larger populations [7],[39]. In the case of binary discrimination, we have shown a *hypothesis of model form* can replace some deep learning computing. This could have implications, for example, for expeditious differentiation of cancerous and non-cancerous cells.

The luxury of large degrees of freedom inherent in the AR-RFR method of image parameter estimation is unique in current MRI brain scan studies and deserves additional attention.

### 5.3 Acknowledgments

WO thanks the UIC department of Biomedical Engineering for the sabbatical that made this article possible. Funding for this work was also provided by the National Institutes of Health (NIH/NCI K22CA258410 and NIH/ NIGMS R35GM151041), both to MD

## References

[1] Gazzaniga, M., 2008. Human. HarperCollins, New York.

[2] Kimura, M and Tanaka, M.,2020, Perspective of interpretability of deep neural networks. 3rd International Conference on Information and Computer Technologies, San Jose, CA, pp. 78-85, doi: 10.1109/ICICT50521.2020.00020.

[3] Prince, G.D.Simon, 2023, Understanding Deep Learning, MIT Press, Cambridge MA.

[4] S. Iqbal, A.N. Qureshi, 2022, A heteromorphous deep CNN framework for medical image segmentation using local binary pattern, IEEE Access, pp.63466-63480.

[5] McLachlan, G. C. 2001, Discriminant Analysis, Wiley Online Library.

[6] Hussain, M., LaMay, D., Grant, E., *et al* 2024, Deep learning of structural MRI predicts, crystalized, and general intelligence, Nature Scientific Reports, pp. 2045-2322.

[7] Thompson, N., Greenewald, K., Lee, K., Manso, G.,2020. The computational limits of deep learning. MIT initiative on the digital economy research brief, Vol.4, pp.1-4.

[8] Rawat, W. and Wang,W., 2017, Deep convolutional neural networks for image classification : A comprehensive review, Neural Computation, pp. 2352-2449.

[9] O'Neill. W. Penny, S. 2019, Image identification of animal tumor models for human breast cancer research. IEEE EMBS, International Conference on Biomedical Health Informatics (BHI), pp.1-4.

[10] van Oostveen, W. and de Lange, E., Imaging techniques in Alzheimer's Disease: A review of applications in early diagnosis and longitudinal monitoring, 2021, Int. J. Mol. Sci., 22, 2110. https://doi.org/ 10.3390/ijms22042110, 34 pp.

[11] McShane, B. and Gal, D. 2017. Statistical significance and the dichotomization of evidence. J. Am. Stat. Assoc. Vol. 112, pp. 885–895.

[12] Cepeda, S. *et al,* 2024, Deep learning-based postoperative glioblastoma segmentation and extent of resection evaluation: Development, external validation, and model comparison, Neuro-Oncology Advances,Vol. 6, Issue 1.

[13] Baltissen, D.,Wollmann,T., Gunkel,M., Chung,I., Erfle,H., Rippe,K., Rohr,K,2018, Comparison of segmentation methods for tissue microscopy images of glioblastoma cells, IEEE 15th International Symposium on Biomedical Imaging (ISBI 2018) April 4-7, 2018, Washington, D.C., US.

[14] Germond, A. *et al* , 2018, Cell type discrimination based on image features of molecular component distribution. *Sci Rep* **8**, 11726.

[15] Ahmed, F., *et al*, 2023, Bioimaging-guided discrimination of normal/cancer cells using $Ag^{+}$-mediated red fluorescent carbon dots. Chem. Eng. Jour. Vol.477, 147300.

[16] Keskin, F.,Suhre, A., Erşahin, T., Cetin, A.,2012, Carcinoma cell line discrimination in microscopic images using unbalanced wavelets*, 2012. 46th Annual Conference on Information Sciences and Systems,* Princeton, NJ, pp. 1-6.

[17] Evered, A., Walker, D., Watt, A., N. Perham. 2013. Untutored discrimination training on paired cell images influences visual learning in cytopathology., Cancer Cytopathology, Vol.122, Issue 3, pp. 200-210.

[18] Wurth, R.,2025. Nature Cancer,6. pp. 67-85.

[19] Garabedian, P.,1964. Partial differential equations, first ed.Wiley and Sons,New York.

[20] Jain, A.,1977, Partial-differential equations and finite difference methods in image- processing J. Opt.Theory. App.Vol. 23, pp. 65-91.

[21] Robertson, C. and George, S. ,2012, Theory and practical recommendations for autocorrelation-based image correlation spectroscopy, J. Biomed. Optics, pp.1-8.

[22] Beard, D. and Bassingthwaighte, J., 2000, Advection and diffusion in substances in biological tissues with complex vascular networks, Annals of Biomedical Engineering, pp. 253-268.

[23] Dehghan, M.,2004. Numerical solution of the three-dimensional advection–diffusion equation., App. Math. and Comp. Vol. 150, pp. 5-19.

[24] Aston, J. and Johansen, A., Bayesian solutions to selected problems in neuroimaging, 2015, Current trends in Bayesian methodology with applications, 18,1.

[25] Arbabshirani, M. 2014, Impact of autocorrelation on functional connectivity. NeuroImage., Vol. 102,Part 2, pp.294-308.

[26] Woods, J., 1972. Two Dimensional discrete Markovian fields., IEEE Trans. Info. Theo., Vol. IT-18, pp. 232-240.

[27] Xun, X., Cao,J., Mallick, B., Maity, A., Carroll, R., 2013. Parameter estimation of partial differential equations., J. Amer. Stat. Assoc., Vol. 108, No. 503. pp. 1009-1020.

[28] Adi Ben-Israel and T. N. E. Greville. Generalized Inverses: Theory and Applications. Wiley and Sons, York, 1974. Second edition, Springer, 2003.

[29] ONeill, W. Penn, R. Werner, M. Thomas, J. 2015. A theory of fine structure image models with an application to detection and classification of dementia. Quan. Imag. Med. Surg. doi: 10.3978/j.issn.2223-4292.2015.03.11. pp. 1-13.

[30] Wilks, S.1963. Mathematical Statistics. Wiley and Sons, New York.

[31] Chan,T., 1984, Stability analysis of finite difference schemes for the advection-diffusion equation., SIAM Journal on Numerical Analysis, Vol.21, No. 2, pp. 272-284.

[32] Khansari, M. O'Neill, W., Lim, J., Shahidi, M., 2017. Method for quantitative assessment of retinal vessel tortuosity in optical coherence tomography angiography applied to sickle cell retinopathy. Biomedical Optics Express., Vol. 8, No. 8. pp. 3796-3806.

[33] Sadeia, B. Munaweera, R. Anderson, J. ONeill, W. Hu, Y. 2021. Rapid statistical discrimination of fluorescence images of T cell receptors on immobilizing surfaces with different coating conditions. Nature: Scientific Reports, (Supplementary material available at https://doi.org/10.1038/s41598-021-94730-3).

[34] White, H. 1980. A heteroskedasticity-consistent covariance matrix estimator and a direct test for heteroskedasticity. Econometrica, Vol. 48, No.4. pp. 817-838.

[35] Kullback, S. 1968. Information Theory and Statistics. Dover, Mineola, New York.

[36] Onwudiwe, K., Najera, J., Datta, M., 2024. Single-cell mechanical assay unveils viscoelastic similarities in normal and neoplastic brain cells. Biophysical Journal, 123(9), pp.1098-1105

[37] Salloway, S., Alber,J., Athene, L., Snyder,P., 2024, Retinal Imaging: The Eye as a Window to the Brain. Carney Institute for Brain Science, Brown University.

[38] Sheu, Y. 2020. Illuminating the black box: interpreting deep neural network models for psychiatric research. Front Psychiatry: Deep learning in Psychiatry. Oct 29; 11:551299 doi: 10.3389/fpsyt. 551299.

[39] Lin, J. *et al*, Screening for cognitive impairment in older adults: A systematic review for the U.S. Preventive Services Task Force. Ann. Intern. Med. 2013;159:601-612.

KEYWORDS and PHRASES

AUTOREGRESSIVE IMAGE MODELS

RANDOM FIELD REPRESENTATIONS

BINARY DEMENTIA DISCRIMINATION

DEGREES OF FREEDOM MODELING

MODEL PARAMETER STATISTICAL SIGNIFICANCE

LIMITED SUBJECT POPULATION

ACCELERATED PARAMETER ESTIMATION

SUGGESTED REVIEWERS

1. TIMOTHY CARROLL, UNIVERSITY OF CHICAGO, PROF. OF RADIOLOGY, TJCARROLL@UCHICAGO.EDU
2. LALEH RAD, ASSOC. PROF. BME, NORTHWESTERN U. RAD1@NORTHWESTERN.EDU
3. MAHNAZ SHAHIDI, PROF. BME, U OF SOUTHERN CALIFORNIA, MSHAHIDI@USC.EDU
4. WIKTOR OLSZOWY, PROF. CLINICAL NEUROSCIENCES, U. OF CAMBRIDGE U.K. OLSZOWYW@GMAIL.COM
5. JOHN ASTON, PROF. STATISTICS LABORATORY, U. OF CAMBRIDGE U.K. J.ASTON@STATSLAB.CAM.AC.UK
6. POLINA GOLLAND, PROF. MIT COMPUTER SCIENCE AND ARTIFICIAL INTELLIGENCE LABORATORY, POLINA@CSAIL.MIT.EDU.
7. PASHA RAZIFAR, PROF. MOLECULAR IMAGING, UPPSALA U. PASHA.RAZIFAR@UPPSALA.IMENET.SE

### AUTHOR CONTRIBUTIONS

WO (woneill@uic.edu), MD (mdatta@nd.edu), and JN (jnajera2@nd.edu) all contributed to writing the MS text.
MD also designed the brain cell experiments.
JN also performed the brain cell experiments.
WO is the corresponding author.

### DATA AND CODE AVAILABILITY

Image data is available from the corresponding author, while the computer code is routine Matlab system identification tool-box files. No custom coding was employed.

### DECLARATION OF COMPETING INTERESTS

None of the authors declare competing interests.

### ARTICLE SIGNIFICANCE AND JOURNAL FIT

The article is completely concerned with image modelling and as such is mainstream science. It is the only article we know of that challenges deep learning (DL) directly in the binary discrimination task and does so several orders of magnitude quicker in time. Expanding to ternary and higher order discriminations does not increase computation time significantly. One practical article contribution is that it allows for small subject classes the use of readily available computer resources to detect pathologies from images. Another contribution is that AR-RFR can produce image parameter sets at video speeds which might be linked to fMRI scans. The cancer cell model affirms the AR-RFR technique for very noisy asymmetric images producing a model with a strongly preferred and limited parameter set. This is a very desirable property for microscopic image models to be used for cancer cell discrimination. The cell model also opens the door to image modeling which exploits the lavish degrees of freedom available in an image and their contribution to the image autocorrelation function.

### RELATED SUBMISSIONS

MRI images are from a previous study [1], but randomly restricted to twelve subjects per class from a total population of 97 subjects. Archive image data and associated restrictions for the twelve subjects were honored.

### AUTHOR AGREEMENT

The authors agree that the article should be published under the CCBY license.

[1] ONeill, W. Penn, R. Werner, M. Thomas, J. 2015. A theory of fine structure image models with an application to detection and classification of dementia. Quan. Imag. Med. Surg. doi: 10.3978/j.issn.2223-4292.2015.03.11. pp. 1-13.
